# Dual-band complex-amplitude metasurface empowered high security cryptography with ultra-massive encodable patterns

**DOI:** 10.1515/nanoph-2024-0314

**Published:** 2024-07-24

**Authors:** Zhen Gu, Rensheng Xie, Haoyang Liu, Yiting Liu, Xiong Wang, Hualiang Zhang, Jianjun Gao, Liming Si, Shuqi Chen, Jun Ding

**Affiliations:** Shanghai Key Laboratory of Multidimensional Information Processing, Key Laboratory of Polar Materials and Devices, 12655 East China Normal University, Shanghai 200241, China; Department of Broadband Communication, Peng Cheng Laboratory, Shenzhen 518108, China; School of Information Science and Technology, ShanghaiTech University, Shanghai 201210, China; The College of Engineering, Computing and Cybernetics, Australian National University, Canberra, ACT 2601, Australia; Department of Electrical and Computer Engineering, University of Massachusetts Lowell, Lowell, MA 01854, USA; Beijing Key Laboratory of Millimeter Wave and Terahertz Technology, School of Integrated Circuits and Electronics, Beijing Institute of Technology, Beijing 100081, China; The Key Laboratory of Weak Light Nonlinear Photonics, Ministry of Education, School of Physics and TEDA Institute of Applied Physics, Nankai University, Tianjin 300071, China

**Keywords:** cryptography, ultra-capacity, dual-band meta-hologram, complex-amplitude modulation, information security

## Abstract

The significance of a cryptograph method lies in its ability to provide high fidelity, high security, and large capacity. The emergence of metasurface-empowered cryptography offers a promising alternative due to its unparalleled wavefront modulation capabilities and easy integration with traditional schemes. However, the majority of reported strategies suffer from limited capacity as a result of restricted independent information channels. In this study, we present a novel method of cryptography that utilizes a dual-band complex-amplitude meta-hologram. The method allows for the encoding of 2^25^ different patterns by combining a modified visual secret-sharing scheme (VSS) and a one-time-pad private key. The use of complex-amplitude modulation and the modified VSS enhances the quality and fidelity of the decrypted results. Moreover, the transmission of the private key through a separate mechanism can greatly heighten the security, and different patterns can be generated simply by altering the private key. To demonstrate the feasibility of our approach, we design, fabricate, and characterize a meta-hologram prototype. The measured results are in good agreement with the numerical ones and the design objectives. Our proposed strategy offers high security, ultra-capacity, and high fidelity, making it highly promising for applications in information encryption and anti-counterfeiting.

## 1 Introduction

Information security is of vital importance in modern wireless communications. Numerous information cryptography techniques have been investigated to attain a high level of security [1], [2], [3], [4], [5]. Among these, the benefits of metasurface-empowered cryptography, which allows for unprecedented manipulation of electromagnetic waves with multiple degrees of freedom (DoFs) (e.g., wavelength, polarization, phase, amplitude, etc.) [6], [7], [8], [9], [10], [11], [12], [13], [14], [15], [16], [17], [18], [19], [20], [21], [22], [23], [24], [25], [26], [27], [28], [29], [30], [31], [32], [33], [34], [35], [36], [37], [38], [39], [40], [41], [42], [43], [44], [45], [46], [47], [48], open up a new path for high security and large capacity. For instance, the wavelength can be used as a key for encryption, and the secret message can be obtained at the predetermined wavelength [6]. Using polarization as the key is another popular tactic [7], [8], [9], [10], [11]. By selecting the preset input and output polarization combinations, a 12-channel metasurface has been demonstrated [7]. Apart from the polarization-multiplexed encryption, the incident field modulated technology has also been employed for encryption [12], [13]. A re-programmable meta-hologram is demonstrated for encryption with a high security level, where the secret message can only be extracted with a specific phase-modulated laser beam [13]. However, the secret message could be decrypted when a hacker intercepts all channel information in the aforementioned encryption methods. To tackle this issue, the metasurface-based encryption techniques that combine several DoFs have gained more attention. The utilization of multiple DoFs can not only greatly enhance the information capacity, but also raise the information security level by making the decryption key more complex [20], [21], [22], [23], [24], [25], [26], [27], [28], [29], [30], [31], [32].

Moreover, the VSS is a common computational imaging scheme that has been employed to metasurface-based cryptographies for improving the security of the secret message [34], [35], [36]. Polarization is used as the key in a theoretical demonstration of a secret-shared encoding technique based on the VSS [34]. In addition, to improve the storage of the encryption method without adding more channels, an encryption scheme by using one-time-pad key is proposed to generate 16 distinct images [35]. Although the security level could be increased by using the VSS-based methods, part of the fidelity is lost in the decrypted results, and the storage capacity is not large enough. In addition, by combining the holographic technology with the single-pixel imaging technology, an encryption method based on spatially multiplexed metasurface is demonstrated to include 26 letters and 10 numbers [33]. However, the number of unique symbols that can be encrypted by this method is determined by the metasurface size. Thus, it is highly demanded to develop a new cryptography approach that can encode a vast number of different symbols without requiring a large number of channels, while also ensuring the security and fidelity of the secret message as well as enabling dynamic adjustment of them.

In this work, we propose a novel cryptography method based on a dual-band complex-amplitude metasurface, which could encode 2^25^ different patterns or symbols. In the encryption phase, a cipher image is generated by extracting the features of the 2^25^ different patterns, which is transformed to the two frequency-selective shared keys (SKs) using the modified VSS scheme and a private key for each symbol in the secret message is converted to a 25-bit intensity sequence. Additionally, the two SKs are transformed to amplitude and phase distributions by the Gerchberg-Saxton (GS) algorithm, which are then recoded into a dual-band complex-amplitude metasurface. During the decryption phase, the two SKs represented by two holographic images can be reconstructed under a circularly polarized incidence, and the private key is received from a wireless channel. A cipher image is reconstructed by first performing an “XNOR” operation on the two SKs, and the secret symbol can be decrypted by further performing an “XOR” operation on the cipher image and the private key. Because each private key corresponding to a distinct symbol, this method is also known as a one-time-pad encryption method, which is theoretically unbreakable. Moreover, different message can be transmitted with the same metasurface by simply alternating the private keys, enabling dynamic adjusting of message. In this work, we only need to transmit the private key (i.e., a 25-bit binary intensity sequence) in the wireless channel. The SK_1_ and SK_2_ are transmitted by the meta-hologram, which can be reconstructed at a pre-set image plane under the correct incidence. By using the modified VSS and the one-time-pad encryption method, the proposed cryptography method features a much higher fidelity and could achieve ultra-massive encodable patterns with a higher security level, showcasing significant potential for advanced data storage and encryption.

## 2 Results and discussion

### 2.1 Operating principle and metasurface design

**Figure 1:**
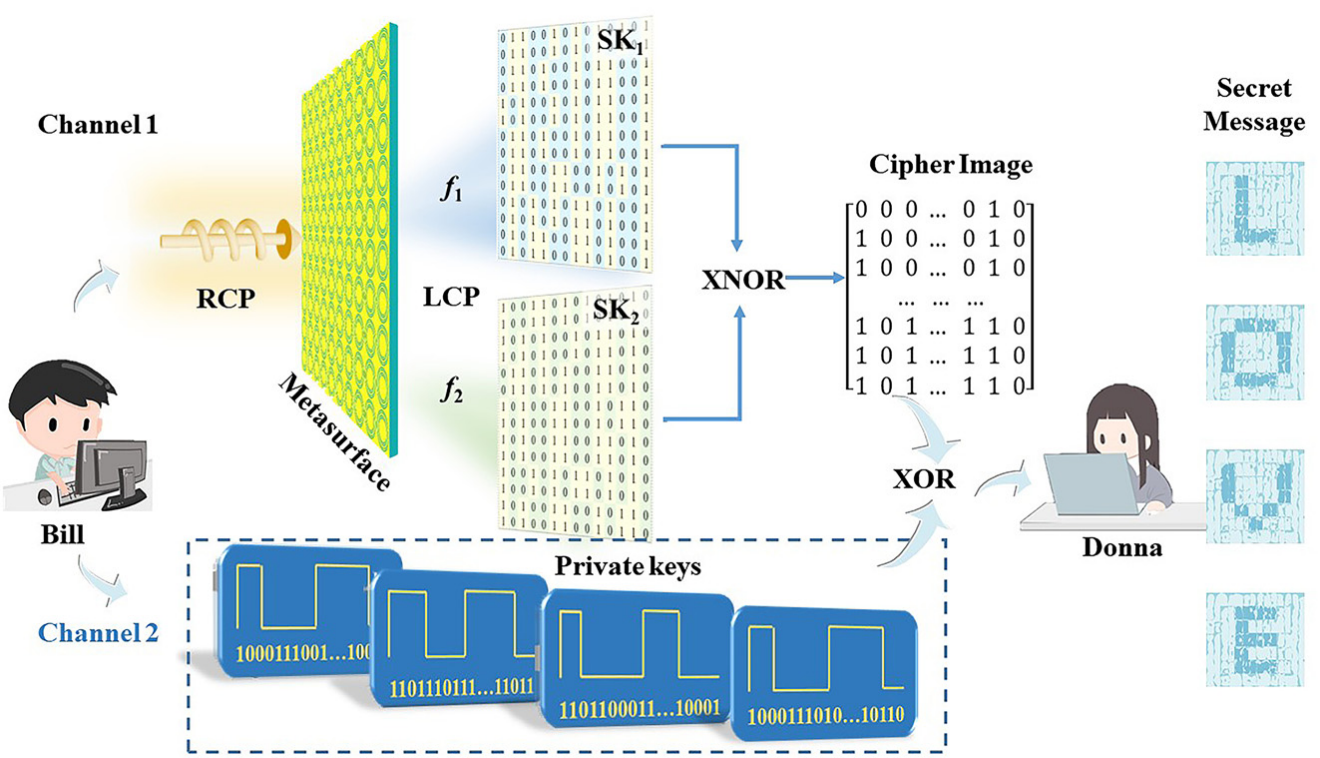
The schematic diagram of the proposed cryptography method.

Figure 1 shows the schematic diagram of the proposed cryptography technique, which assumes that Bill wants to send a message to Donna. At the transmitting end, two SKs are encoded into a dual-band metasurface with complex-amplitude modulation. In addition, each symbol in the message is transformed into a private key as a 25-bit binary intensity sequence, which is then sent from a wireless channel. Moreover, this process so called “one-time-pad” encryption method allows the encoded message to be modified simply by altering the transmitted private keys, which could unbreakable [35]. At the receiving end, two holographic images SK_1_ and SK_2_ are reconstructed at frequencies *f*_1_ and *f*_2_, respectively, and an oscilloscope is used to receive the intensity signals (i.e., private keys). Then, Donna can superimpose the two SKs to generate the cipher image, and then decipher the message symbol by symbol through superimposing the private key sequence according to the decoding mechanism. Moreover, even if both the SKs and the private keys are intercepted (note: this is difficult due to the different encoding mechanisms and transmitting channels), the message cannot be deciphered without obtaining the decoding mechanism.

**Figure 2:**
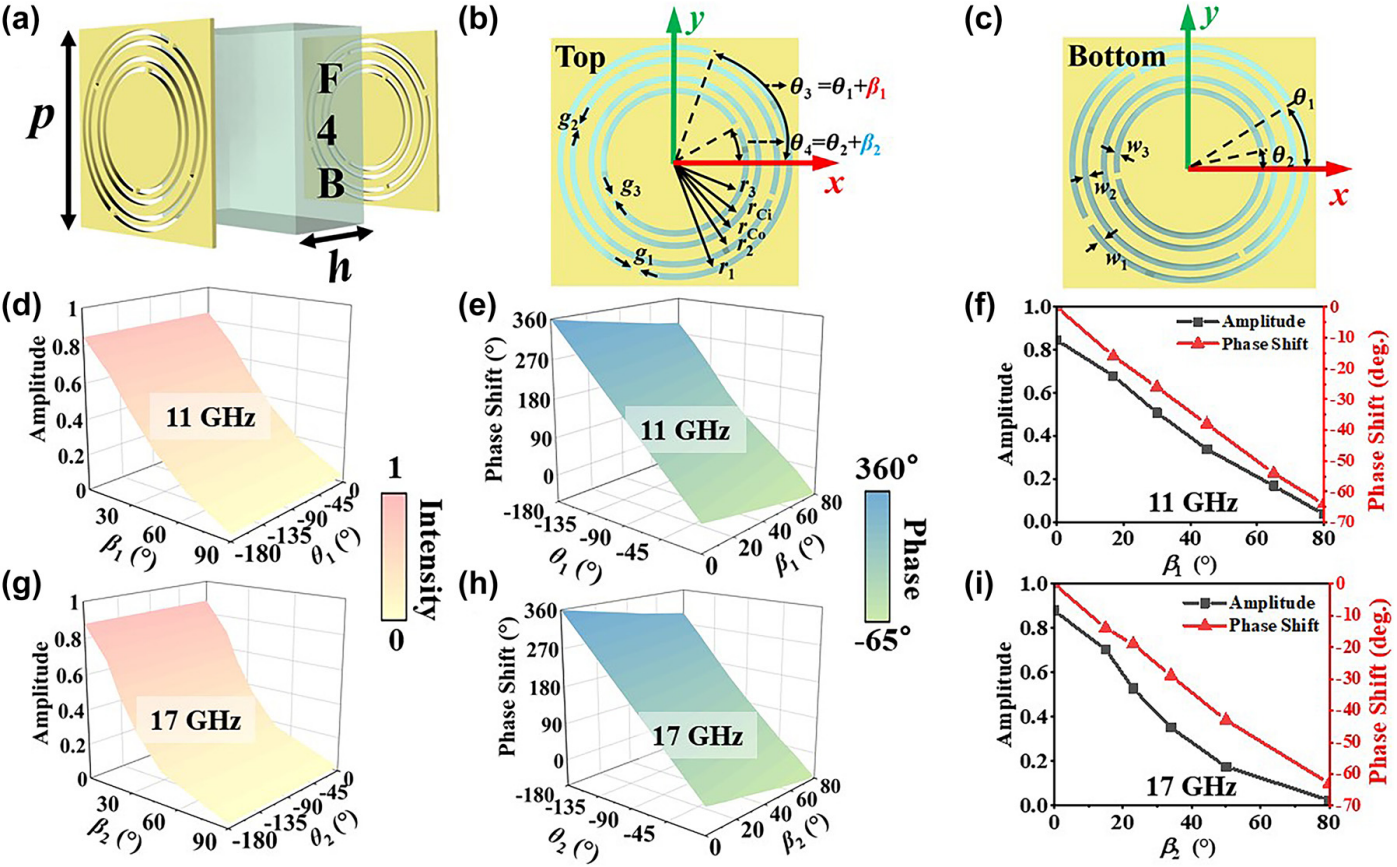
The proposed complex-ampliude meta-atom and their simulated amplitude and phase response. (a) The three-dimensional view, (b) top view, and (c) bottom view of the proposed complex-amplitude meta-atom. Specifically, *p* = 8, *h* = 4, *g*_1_ = 0.3, *g*_2_ = 0.2, *g*_3_ = 0.2, *w*_1_ = 0.2, *w*_2_ = 0.2, *w*_3_ = 0.2, *r*_1_ = 3.6, *r*_2_ = 3.2, *r*_Co_ = 2.8, *r*_Ci_ = 2.6, *r*_3_ = 2.2 (unit: mm). (d) The amplitude and (e) phase shift compared with (0°, 0°) orientated meta-atom as functions of *β*_1_ and *θ*_1_ are presented at 11 GHz. (f) The amplitude and phase response with varied rotation angle *β*_1_ at 11 GHz. (g) The amplitude and (h) phase shift compared with (0°, 0°) orientated meta-atom as functions of *β*_2_ and *θ*_2_ are presented at 17 GHz. (i) The amplitude and phase with varied rotation angle *β*_2_ at 17 GHz.

As an illustrative example, Figure 2(a) depicts a dual-band complex-amplitude meta-atom, which is composed of two metallic layers printed on opposing surfaces of an F4B dielectric substrate (*ε*_r_ = 2.2, tanδ = 0.001). Figure 2(b) and (c) plot the top and bottom metallic layers, consisting of a modified complementary split-ring resonator (MCSRR) and a modified double-C-slot resonator (MDCSR) located at the center. It is worth mentioning that the top and bottom layers have identical physical dimensions except for the orientation angles of the resonators. The orientation angles of the MCSRR and MDCSR denoted as *θ*_1_ (*θ*_3_) and *θ*_2_ (*θ*_4_) on the bottom (top) layer are defined as the angle between their incision gaps and the *x*-axis, respectively. The orientation angle difference between the MCSRRs (MDCSRs) on the top and bottom metallic layers is denoted as *β*_1_ = *θ*_3_ − *θ*_1_ (*β*_2_ = *θ*_4_ − *θ*_2_). During the meta-atom design process, full-wave simulations were conducted by employing CST Microwave Studio, where the unit-cell boundary conditions were applied in both the *x*- and *y*-directions. The meta-atom was illuminated under a normal right-handed circularly polarized (RCP) incidence, and the phase and amplitude responses can be extracted by recording the transmitted left-handed circularly polarized (LCP) wave.

The simulated amplitude and phase responses by varying *β*_1_ and *θ*_1_ at 11 GHz are plotted in Figure 2(d) and (e), respectively. Figure 2(d) shows that the continuous amplitude modulation from 0 to a maximum, while Figure 2(e) demonstrates the whole 2*π* phase modulation. It can be seen from Figure 2(d) that the amplitude only depends on *β*_1_, which changes very little when *θ*_1_ is varied. Moreover, Figure 2(e) illustrates that a little phase shift might be induced by varying either *β*_1_ or *θ*_1_. Similar conclusions can be drawn from Figure 2(g) and (h) at 17 GHz, indicating that the amplitude response depends only on *β*_2_, and the phase response depends on both *β*_2_ and *θ*_2_. In general, both the amplitude and phase modulation can be realized at two pre-set frequencies by varying the *θ*_1_ (*θ*_2_) and *β*_1_ (*β*_2_). In addition, the phase controls of the proposed meta-atom at two operating frequencies are completely independent, which is shown in Section I in the Supporting Information. Furthermore, the simulated amplitude and phase responses by varying *β*_1_ (*β*_2_) at 11 GHz (17 GHz) are plotted in Figure 2(f) and (i), respectively, where *θ*_1_ (*θ*_2_) is fixed to be 0°. It can be observed that the amplitude varies from 0.88 (0.85) to 0 by increasing *β*_1_ (*β*_2_) from 0° to 80° at 11 GHz (17 GHz). The theory analysis of the complex-amplitude modulation can be found in Section I in the Supporting information. In addition, the phase response decreases as *β*_1_ or *β*_2_ increases, which can be compensated by varying the *θ*_1_ (*θ*_2_) with an additional value also detailed in this part.

### 2.2 Encryption method

**Figure 3:**
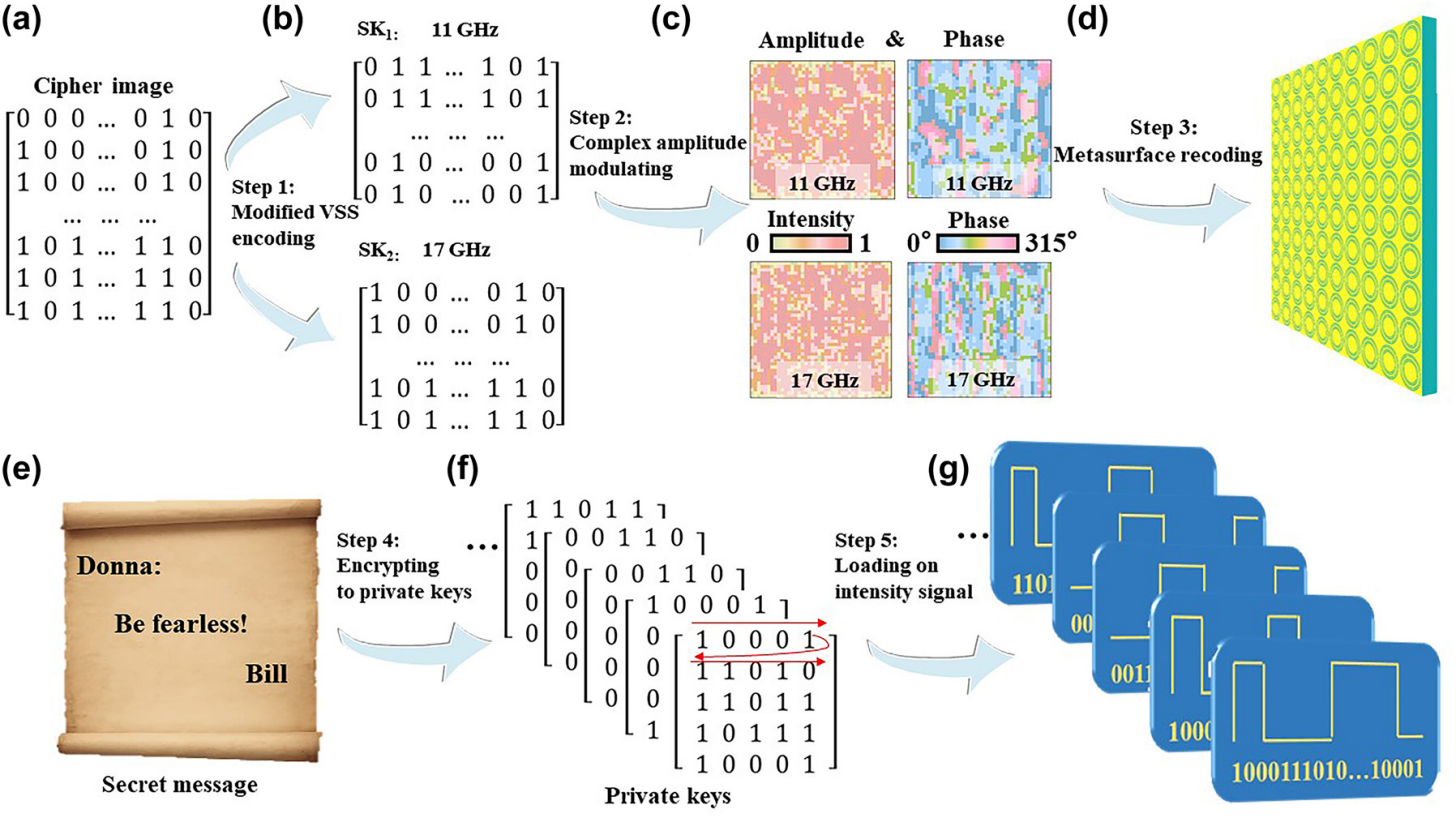
The process of the proposed cryptography method based on the dual-band complex-amplitude metasurface. (a) The cipher image. (b) The shared keys (SKs). (c) The optimized amplitude and phase patterns for the SKs. (d) The dual-band complex-amplitude metasurface. (e) The secret message. (f) The corresponding private keys. (g) The intensity signal loaded private keys.

The encryption procedure is illustrated in Figure 3(a)–(g), which includes five key steps: the modified VSS encoding, complex-amplitude modulating, metasurface recoding, encrypting to the private keys, and loading on the intensity signals. In order to achieve a large capacity, we select a 5 × 5 matrix to represent a distinct symbol, whereby a white pixel (black pixel) of the matrix element is assigned a value ‘1’ (‘0’). In this way, a total of 2^25^ different symbols can be encoded. Then, the features of the 2^25^ different patterns are extracted to create a cipher image as a 7 × 7 matrix, which is subsequently converted to the two frequency-selective SKs using the modified VSS scheme depicted in Figure 3(a) and (b). In order to avoid the edge loss of the reconstructed hologram affecting the decrypted result, only the middle 5 × 5 part of the 7 × 7 matrix in the cipher image contains the secret message. Moreover, the detailed comparison of the traditional and modified VSS schemes is demonstrated in Section II in the Supporting Information, which indicates a significantly higher level of fidelity for the modified VSS scheme. Besides, it can be seen from Figure 3(b) that no single SK can be used to disclose the whole secret message, which could enhance the security. Considering the SKs are images composed of white pixel ‘1’ and black pixel ‘0’, as many pixel values as possible should be discretized during the complex-amplitude modulation process to better reconstruct the SKs. The details about the how to design SK_1_ and SK_2_ could be seen in Section II the supporting information. It is found that the edge characteristics of the SKs can be well reappeared when the pixel corresponds to 3 × 3 or more units. Following the optimization process, the edge characteristics of the pixel can be well reconstructed by the dual-band metasurface consisting of 42 × 42 meta-atoms. It is worth mentioning that the results can be further improved by employing a larger metasurface array. In addition, 3-bit phase modulation and six-level amplitude modulation are adopted. Compared to the traditional phase-only encoding method, the complex-amplitude modulation method could improve the quality of the reconstructed holographic SKs to further avoid fidelity loss in the decrypted results [49].

Moreover, the employed private key is also composed of a 5 × 5 matrix illustrated in Figure 3(f), which is a crucial component of the encryption process. By altering any one of these matrix elements, a new pattern can be produced, resulting in a possible total of 2^25^ unique patterns. Then, the complex-amplitude modulated method is used to hide the SKs into the amplitude and phase distributions displayed in Figure 3(c) by the GS algorithm, which are then recoded into a dual-band metasurface shown in Figure 3(d). Moreover, the transmitter looks up the corresponding private keys according to the secret message (shown in Figure 3(e)) by symbol in the private key dictionary shown in Figure 3(f). Subsequently, the private keys are loaded on the intensity signals to be transmitted shown in Figure 3(g). In this way, a large number of private keys can be transmitted simultaneously in parallel if required. By combining the two frequency-selective SKs, complex-amplitude modulation, and one-time-pad private key, the proposed method could achieve ultra-massive capacity with a much higher-level security and fidelity in comparison to traditional schemes.

### 2.3 Decryption method

The decryption process can be divided into three steps. First, the two SKs (i.e., the two holographic images) are reconstructed at the pre-set image plane, and the intensity signals as the private keys are received from wireless channel. Next, the SKs are superimposed to form a cipher image by applying an “XNOR” operation. Then, each symbol in the message can be decrypted by performing an “XOR” operation on the cipher image and the private key.

**Figure 4:**
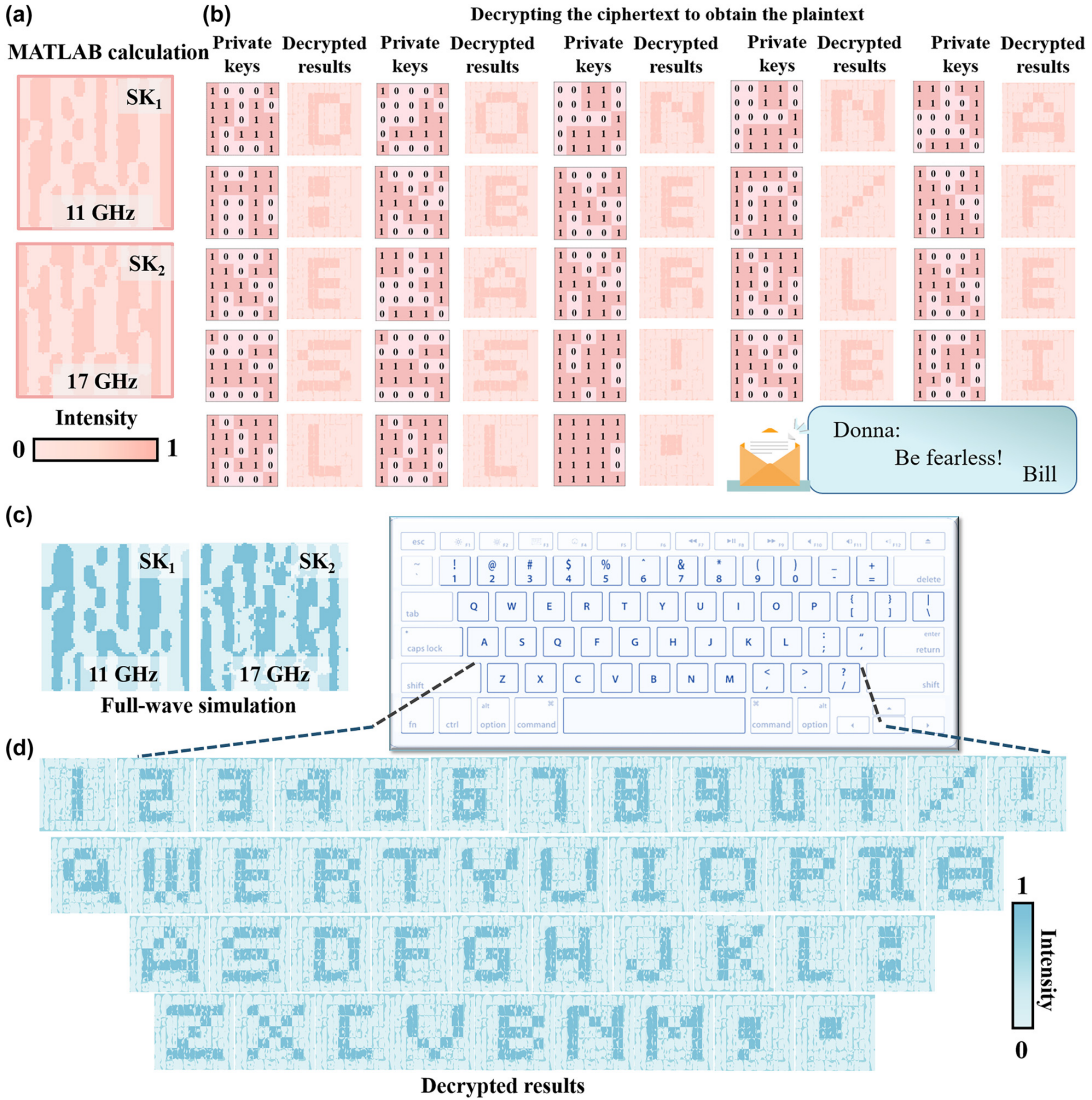
The shared keys (SKs) of MATLAB calculations and full-wave simulations, and their corresponding decrypted results. (a) The SKs calculated by MATLAB. (b) The private key and the corresponding decrypted results (Donna: Be fearless! Bill. “/” represents space, “.” indicates the end of the secret message). (c) The full-wave simulations of the SKs, and (d) the corresponding decrypted results.

The feasibility of the proposed encoding scheme is firstly verified by numerical calculations and full-wave simulations. Figure 4(a) shows that the numerically calculated SKs can be clearly observed at two pre-set frequencies, which are consistent with the original ones in Figure 3(b). Additionally, a message of “Donna: Be fearless! Bill” is used to demonstrate the capability of the proposed encryption method. The private keys and the corresponding decrypted results for each symbol are depicted in Figure 4(b). The period “⋅”means the end of the message. If the decrypted results do not contain this symbol, the transmitted message is incomplete and must be retransmitted. Moreover, the key dictionary consisting of private keys and their corresponding decrypted results calculated by MATLAB is shown in Figure S3 in the Supporting Information. Remarkably, different message can be generated by only changing the private keys that are loaded onto the intensity signals, enabling the secret message to be dynamically adjusted from the decryption end.

### 2.4 Experiment and discussion

In the full-wave simulations, the SKs at two pre-set frequencies could be extracted on the image plane under an RCP plane wave incidence in the CST Microwave Studio. The observation plane is located at a distance of *d* = 100 mm away from the metasurface and parallel to the metasurface. Figure 4(c) plots the two simulated SKs, which are in good agreement with the calculated ones in Figure 4(a). In addition, Figure 4(d) depicts the decrypted results with the provided private keys, which contain 26 letters, 10 numbers, and some special symbols. Noteworthy, this cryptography can theoretically encrypt 2^25^ different patterns, which facilitates the transmission of a greater volume of more intricate information while mitigating potential ambiguities during information dissemination. Some discrepancies between the theoretical calculations and the simulations might be caused by non-ideal edge diffraction of the metasurface in the full-wave simulations and losses in amplitude and phase quantization.

**Figure 5:**
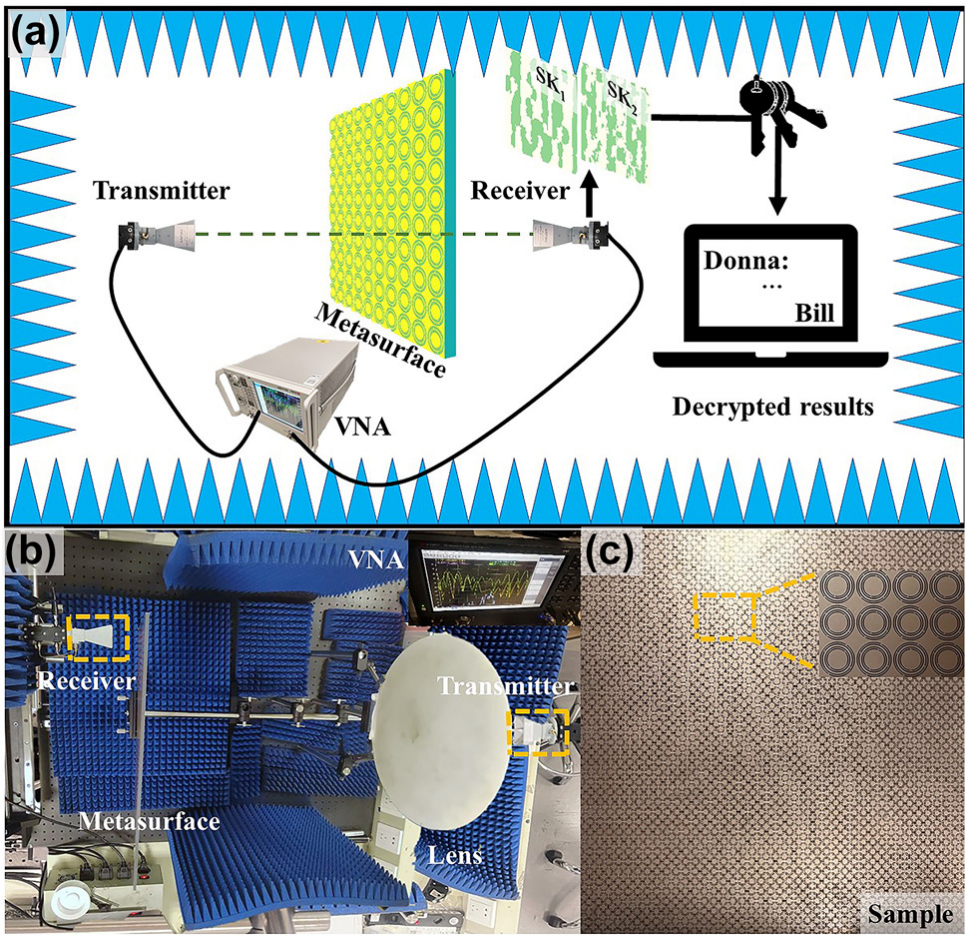
The experimental setup and the fabricated metasurface prototype. (a) The schematic diagram of the experimental setup for decoding the secret message. (b) The photograph of the experimental setup. (c) The fabricated metasurface prototype.

To experimentally validate the proposed method, the designed dual-band metasurface is fabricated and measured. The experimental setup for near-field measurement is illustrated in Figure 5(a). To cover the operating frequencies of 11 and 17 GHz, a pair of dual circularly polarized horn antennas (LB-SJ-60180-P03, 6–18 GHz) was employed as the transmitter and receiver. A quasi-plane wave can be generated by placing a dielectric lens in front of the transmitter, which was then illuminated onto the metasurface sample as shown in Figure 5(b). To measure the transmitted fields, the receiver connected to a vector network analyser (Keysight Technologies, N5227B) was moved with a step of 2 mm in both *x*- and *y*-directions on the image plane at a distance of *d* = 100 mm from the metasurface. Figure 5(c) shows the metasurface sample composed of 42 × 42 meta-atoms with an overall dimension of 336 mm × 336 mm, which was fabricated by the standard printed circuit board (PCB) fabrication process.

**Figure 6:**
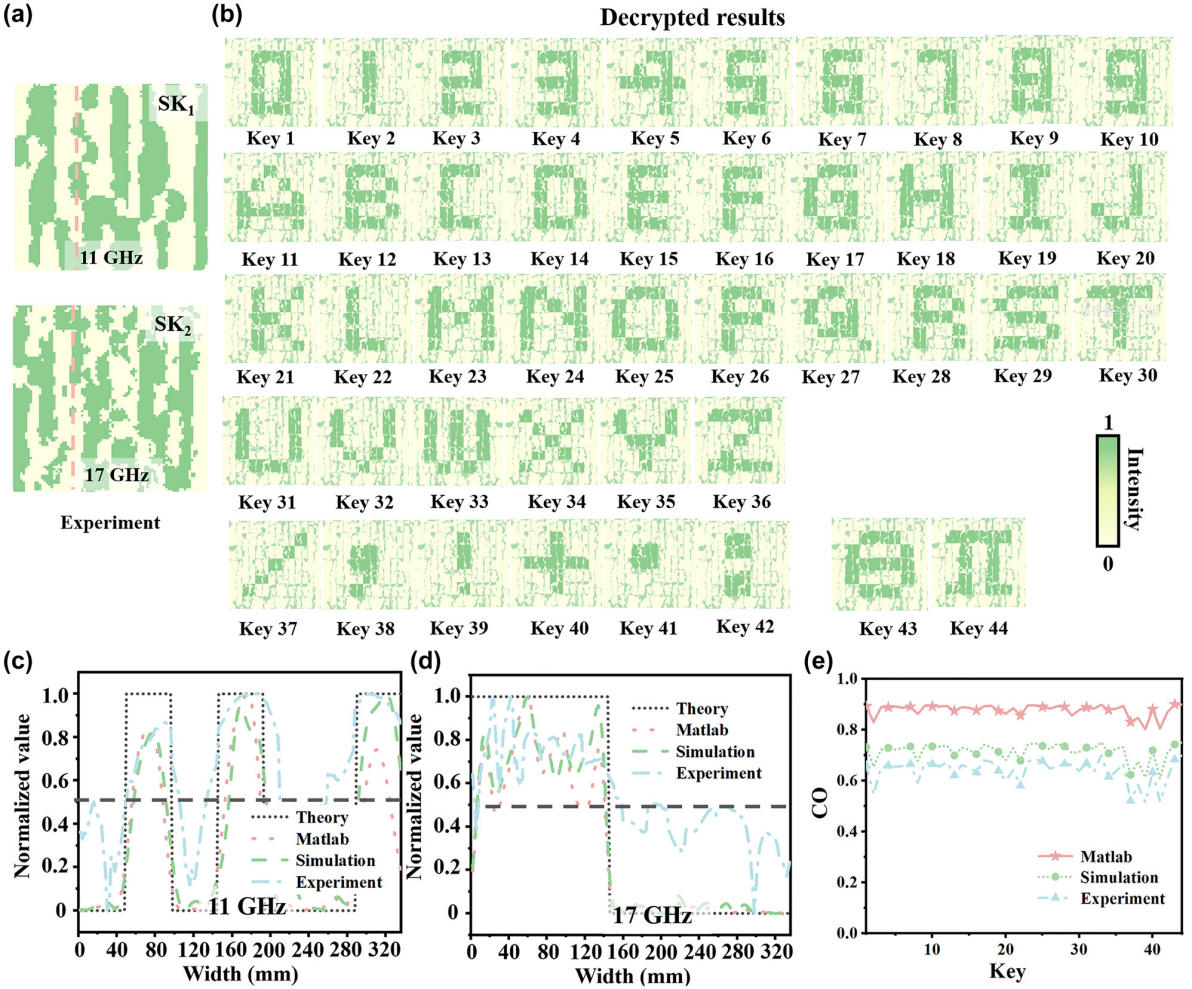
The measured results and the comparisons. The measured results of (a) the SKs and (b) the corresponding decrypted results. The comparison of the (c) SK_1_ and (d) SK_2_ fields obtained by the diffraction theory, MATLAB calculation, full-wave simulation, and experimental measurement. (e) The correlation coefficients (COs) obtained by the MATLAB calculation, simulation and experiment results of decrypted results under the extraction of 44 different private key sequences.

Figure 6(a) plots the measured SKs at the two operating frequencies, which are in good consistency with the calculated and simulated ones depicted in Figure 5(a) and (c). The quality of the measured SKs could be impacted by several factors including the fabrication tolerance, losses in amplitude and phase quantization, and the measurement tolerance. It is noteworthy that each shared unit in the reconstructed SKs should have a significant marginalization feature without obvious distortion between adjacent units in order to achieve complete decryption of the message. The comparison of the cross sections of the electric fields for two reconstructed SKs obtained by numerical calculation, full-wave simulation, and experimental measurement, are conducted. The normalized electric fields are collected at the cross sections of Figure 6(a) along the pink dashed lines. It is evident from Figure 6(c) and (d) that the SKs with various bit values of 1 and 0 can be identified very well. In the MATLAB calculations, each unit cell is simplified as an ideal dipole with the corresponding phase and amplitude, which could provide a much faster numerical calculation of the meta-hologram compared to the full-wave simulations. Additionally, it is noted that the SKs have well-defined edge details due to the complex-amplitude modulation, which is very helpful for subsequent decryption processes.

Figure 6(b) presents the decrypted results of 10 numbers, 26 letters, and some special symbols obtained by the decryption process. It can be observed from Figure 6(b) that the decrypted results can be well recognized and agree very well with the design objects despite of a little decreased quality. Moreover, Figure 6(e) plots the correlation coefficients (COs) between the decrypted results and the original message, which is used to quantitatively evaluate the decoding quality of the decrypted patterns. Both numerical calculation and full-wave simulation results show higher values, verifying higher decoding qualities of the secret patterns. The COs from experiments are relatively low due to the errors in sample processing and experimental testing. In particular, the ideal plane wave in the simulation is configured to impinge directly on the metasurface, whereas the incident wave from the horn antenna in the measurement is not an ideal plane wave. Even though there are some distortions and noises in the decrypted results, the contrast of these results remains discernible to naked eyes. Overall, the revealed parameters verify the capabilities of the proposed cryptography for encoding ultra-massive distinct patterns and guaranteeing a high fidelity of decrypted results simultaneously.

### 2.5 Security and capacity analysis

In the field of cryptography, the security and fidelity are of vital importance. Due to the direct holographic imaging used for the decoding process in traditional metasurface-empowered cryptography, there is a risk of information leakage when a single message is encoded into a single channel. To tackle this issue, the VSS scheme is adopted to hide the secret message into multiple SKs, significantly enhancing the security of the cryptography. However, some information of the decryption result cannot be fully recovered in the VSS scheme as shown in Figure S2(b). To overcome this, a modified VSS scheme is used, unlocking new possibilities for the metasurface-empowered cryptography with a higher fidelity. Moreover, the private key is used to promise for a high security cryptography. Even if the eavesdropper obtains the SKs, the secret message cannot be cracked without the private key. Moreover, even when the SKs and private key are known, the secret message cannot be obtained without knowing the superposition logic of the SKs and the private keys, which provides a new avenue for high-security cryptography.

For metasurface-empowered cryptography, the coverage of the content is as important as security. In traditional metasurface-empowered cryptography, the number of secret messages depends on the number of channels of the metasurface. However, due to the nearly exhausted available channels for information multiplexing, it becomes very challenging to send more information. To overcome this restriction, the proposed metasurface-empowered cryptography can encode a total of 2^25^ different patterns by using 25-bit binary string as the private key, greatly increasing the coverage of secret information. Moreover, the 2^25^ patterns are independent of each other since we encoded the private key into sets of intensity signals. Furthermore, we can send various encrypted message using the same metasurface by simply altering the private keys, which is also known as the “one-time-pad” encryption method. Such a strategy breaks the limitation of the storage on metasurfaces, providing a novel platform for large-coverage encryption and dynamically adjusting secret message.

## 3 Conclusion

In summary, we propose a novel cryptography method based on two frequency-selective SKs generated from a complex-amplitude metasurface and the one-time-pad private keys. Firstly, the features of ultra-massive different patterns (a total of 2^25^ in this work) are extracted as a cipher image and converted into the SKs by the modified VSS scheme, which could heighten the fidelity of the decrypted result. Moreover, the two SKs are encoded into the amplitude and phase distributions based on the iterative GS algorithm and recoded into a dual-band meta-hologram with complex-amplitude modulation, aiming to improve the decryption quality. During the decryption phase, two SKs can be reconstructed at two pre-set frequencies under a RCP incidence, and the private key represented by a set of 25-bit intensity signals can be received from wireless channel. Afterwards, the two SKs are superimposed in accordance with the decoding mechanism of the modified VSS scheme to create a cipher image. Each symbol in the secret message can be then decrypted by performing an “XOR” operation on the cipher image and each private key. In this way, the secret message cannot be deciphered by either the SKs or the private keys. To experimentally verify the proposed approach, a meta-hologram was fabricated and measured, and the measured results demonstrate good agreement with numerical ones and targets. By combining the SKs and different private keys, the proposed metasurface-empowered cryptography can significantly improve the security and the capacity of the secret patterns, offering a new promising route for encryption. Furthermore, by simply changing the private keys or the intensity signal sequences, the transmitted message can be dynamically alternated with the same metasurface.

## Supplementary Material

Supplementary Material Details
