# Supplementary material for: Dual-band complex-amplitude metasurface empowered high security cryptography with ultra-massive encodable patterns
Source: Nanophotonics. 2024 Jul 24;13(20):3915–24. doi: 10.1515/nanoph-2024-0314 (PMC11465982; doi:10.1515/nanoph-2024-0314)
Supplement: Supplementary file 1 — Supplementary Material Details [file j_nanoph-2024-0314_suppl_001.docx]

Supporting Information for:

Dual-band Complex-amplitude Metasurface Empowered High Security Cryptography with Ultra-massive Encodable Patterns

*Zhen Gu^1†^, Rensheng Xie^2†^, Haoyang Liu^3^, Yiting Liu^4^, Xiong Wang ^3^, Hualiang Zhang ^5^,* *Jianjun Gao ^1^, Liming Si^6*^, Shuqi Chen^7*^, Jun Ding^1*^*

^1^Shanghai Key Laboratory of Multidimensional Information Processing, Key Laboratory of Polar Materials and Devices, East China Normal University, Shanghai 200241, China.

^2^Department of Broadband Communication, Peng Cheng Laboratory, Shenzhen 518108, China.

^3^School of Information Science and Technology, ShanghaiTech University, Shanghai 201210, China.

^4^The College of Engineering, Computing and Cybernetics, Australian National University, Canberra ACT 2601, Australia.

^5^Department of Electrical and Computer Engineering, University of Massachusetts Lowell, Lowell MA 01854, U.S.A.

^6^Beijing Key Laboratory of Millimeter Wave and Terahertz Technology, School of Integrated Circuits and Electronics, Beijing Institute of Technology, Beijing 100081, China.

^7^The Key Laboratory of Weak Light Nonlinear Photonics, Ministry of Education, School of Physics and TEDA Institute of Applied Physics, Nankai University, Tianjin 300071, China.

Section I: The theory analysis of the complex-amplitude modulation.

Typically, the incident electric field can be decomposed into two orthogonal components in the *x*- () and *y*- () directions. When the incident waves are linear waves, the transmitted electric fields can be expressed as

(1)

where the matrix *T*_L_ represents the relation between the incident and the transmitted electric fields. Specifically, *T*_xx_ and *T*_yy_ represent the co-polarization transmission coefficients under *x*- and *y*-polarized incidences, respectively. With the same scheme, the transmitted right and left circularly polarized electric fields (i.e., ) under the RCP/LCP illuminations (i.e., ) can be described as [1]:

(2)

where the matrix *T*_C_ represents the relation between the incident and the transmitted electric fields in the circular polarization basis, and *T*_RL_ and *T*_LR_ represent the cross-polarization transmission coefficients under the LCP and RCP incidences, respectively. In addition, *θ* is the orientation angle of the MCSRR or MDCSR on the bottom layer, which means *θ*=*θ*_1_ or *θ*_2_, while *β* is the orientation difference between two MCSRRs or MDCSRs on the top and bottom layers, which indicates *β*=*β*_1_ or *β*_2_. The intermediate variable a is defined as , where and are the impedances of the corresponding polarization states. Therein, the denote the permeabilities and permittivity along the corresponding principal axes. Moreover, . Taking the MCSRR as an example, the *T*_LR_ can be further simplified to according to Equation (2). Theoretically, the *T*_LR_ tends to disappear at *β*_1_ = 90◦, which is the configuration where the principal axes of the two layers are perpendicular to each other. When *β*_1_ = 0°, the system reduces to the initial half-wave plate with a maximum *T*_LR_. Moreover, the 0 to 2π phase modulation can be obtained through by rotating the MCSRR of double layer as a whole. In addition, there is a phase shift of -*β* introduced by the amplitude modulation which is contributed by the geometric transformation process along with the orientation difference of the two layers. As shown in Fig. S1, we change the 𝜃_1_ to counteract the phase shift introduced by varying *β*_1_. The specific value of the 𝜃_1_ is shown in the scale bar. It can be observed from Fig. S1 a that the amplitude are held almost constant while varying the 𝜃_1_. In addition, the 2π phase coverages at 11 GHz can be obtained by simply rotating the 𝜃_1_. Moreover, it can be observed from Fig. S1 that the rotation of the 𝜃_1_ has no effect on amplitudes and phase shifts at another operation frequency. Similar conclusions can be drawn from Figs. S1(g-l) at 17 GHz, indicating that the amplitude and phase controls of the proposed meta-atom at two operating frequencies are completely independent, and the phase shift introduced by varying the *β*_1_ can be counteracted by changing the 𝜃_2_ at 17 GHz.


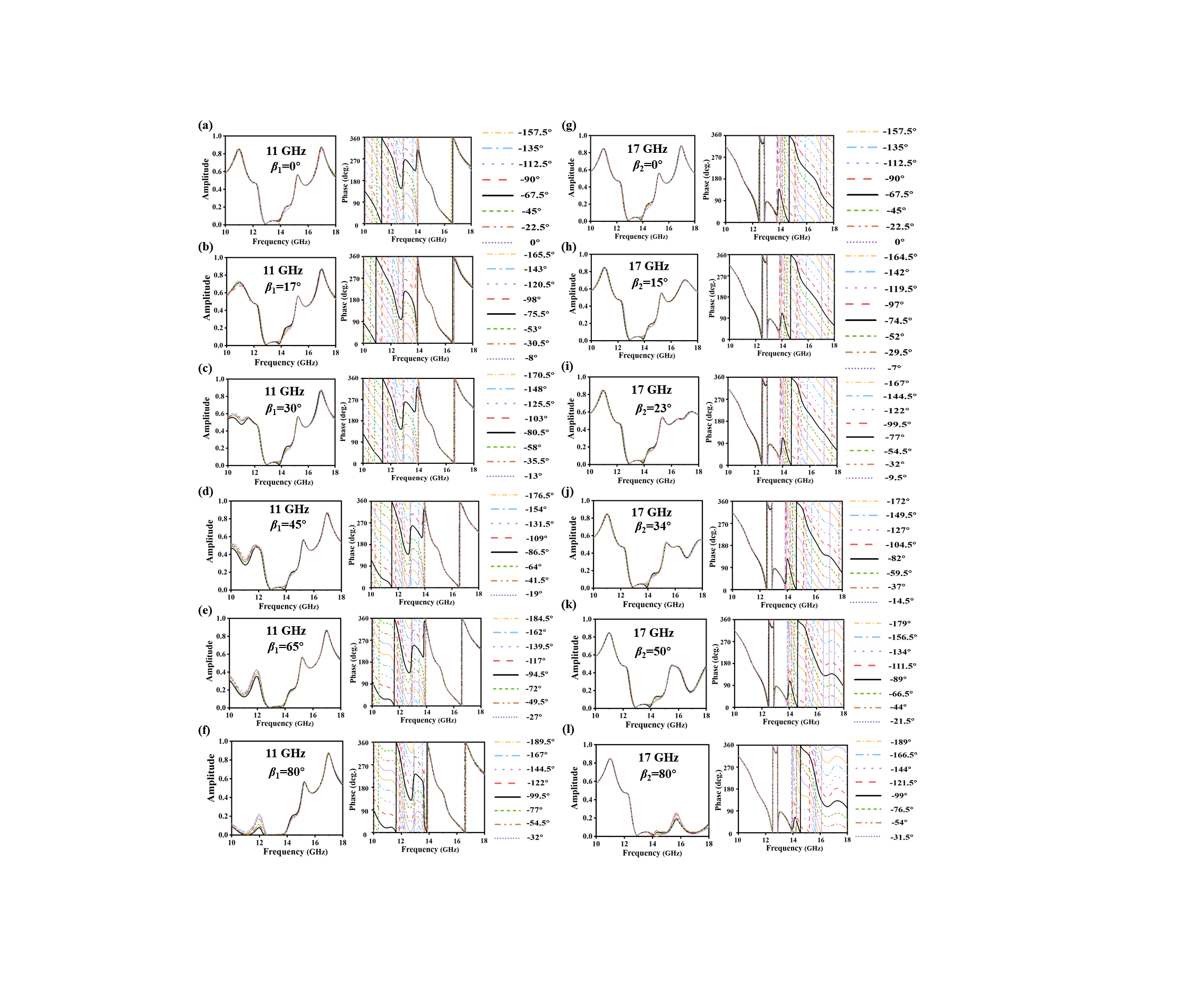


**Figure S1.** (a-f) The amplitudes at 11 GHz while varying the rotation angles of *β*_1_ and the corresponding phase shift when rotating 𝜃_1_ at this *β*_1_. (g-l) The amplitudes at 17 GHz while varying the rotation angles of *β*_2_ and the corresponding phase shift when rotating 𝜃_2_ at this *β*_2_.

Section II. The comparison between the traditional VSS and modified VSS scheme.


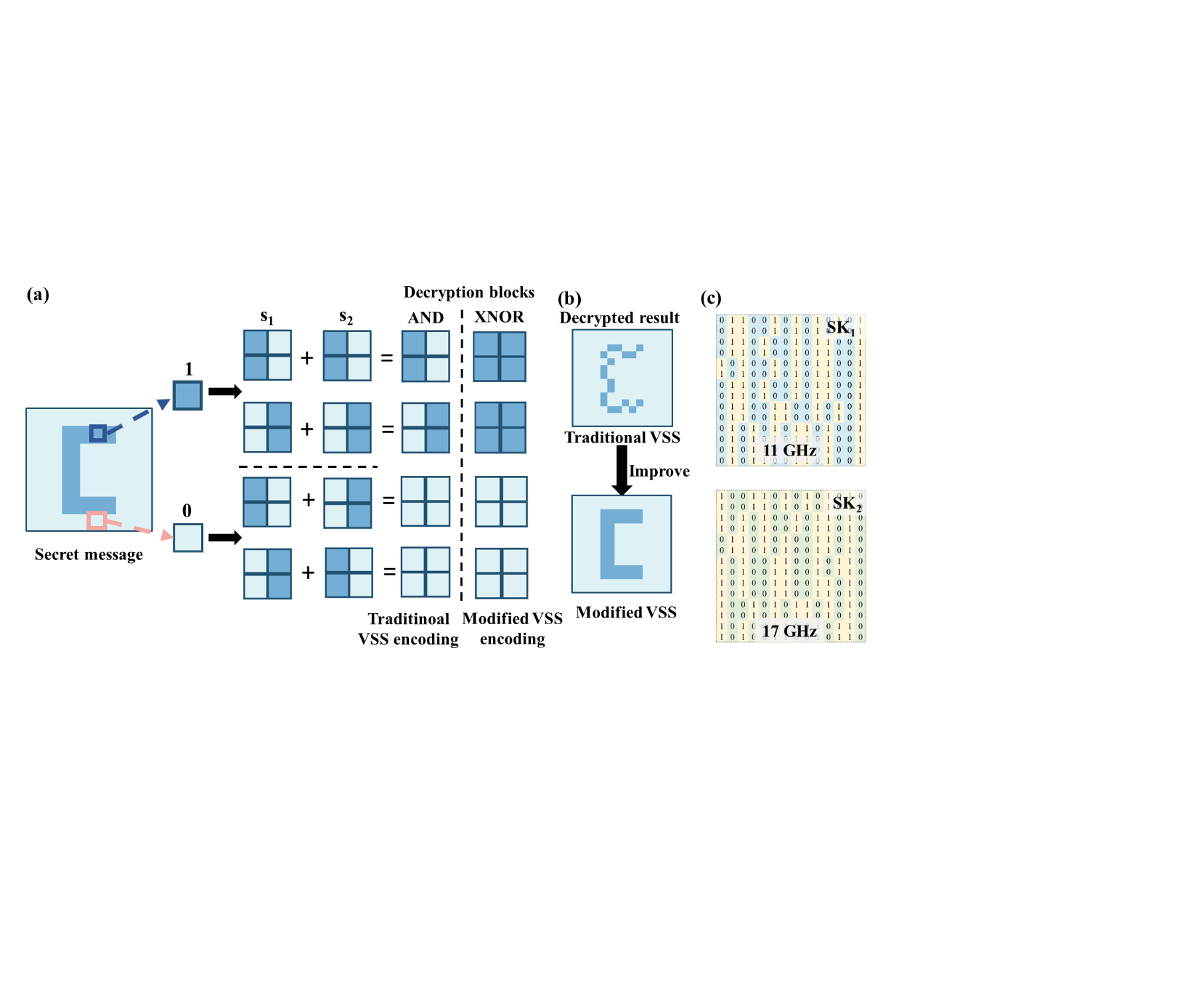


**Figure S2**. (a) The schematic diagram comparison between the traditional VSS scheme and modified VSS scheme. (b) The decrypted results of the traditional VSS scheme and modified VSS scheme. (c) The SKs.

In our method, the modified VSS is employed to generated the SKs. The traditional (2,2) VSS scheme could prevent hackers from deciphering secret message directly by hiding the secret message into two SKs [2-6]. Specifically, the secret pixels in the secret message are encoded into 2 × 2 shared units (*s*_1_ or *s*_2_), and then *s*_1_ and *s*_2_ are used to form SK_1_ and SK_2_, respectively. Fig. S2(a) shows that when the secret pixel is a white pixel ‘1’ (a black pixel ‘0’), the two shared units s_1_ and s_2_ are the same (different). Moreover, the shared unit at different locations are randomly selected and independent of each other, resulting in completely meaningless spatial arrangements of the SKs, which could increase the security of the secret message. The stacking mechanism in the traditional VSS scheme follows the operation “AND”, resulting in half of the decryption blocks corresponding to the while pixel (‘1’) are different from the original ones with a lower fidelity. In our proposed scheme, the stacking mechanism is modified to employ the operation "XNOR", which could completely recover both the black and white pixels and prevent any fidelity loss in the traditional one as depicted in Fig. S2(b). The fidelity of the decrypted results could be defined as the correlation coefficient between the secret image (*S*) and the decrypted result (*D*), which is expressed as [7]

(3)

where *E*[·] denotes the expectation value. Based on Equation (3), the fidelities of the four letters "E," "C," "N," and "U" in the modified VSS scheme are increased to 1 from 0.6815, 0.6704, 0.6508, and 0.6609, respectively, in the traditional VSS scheme. The SKs used in our proposed cryptography are shown in Fig. S2(c).

Section III. The private key dictionary.


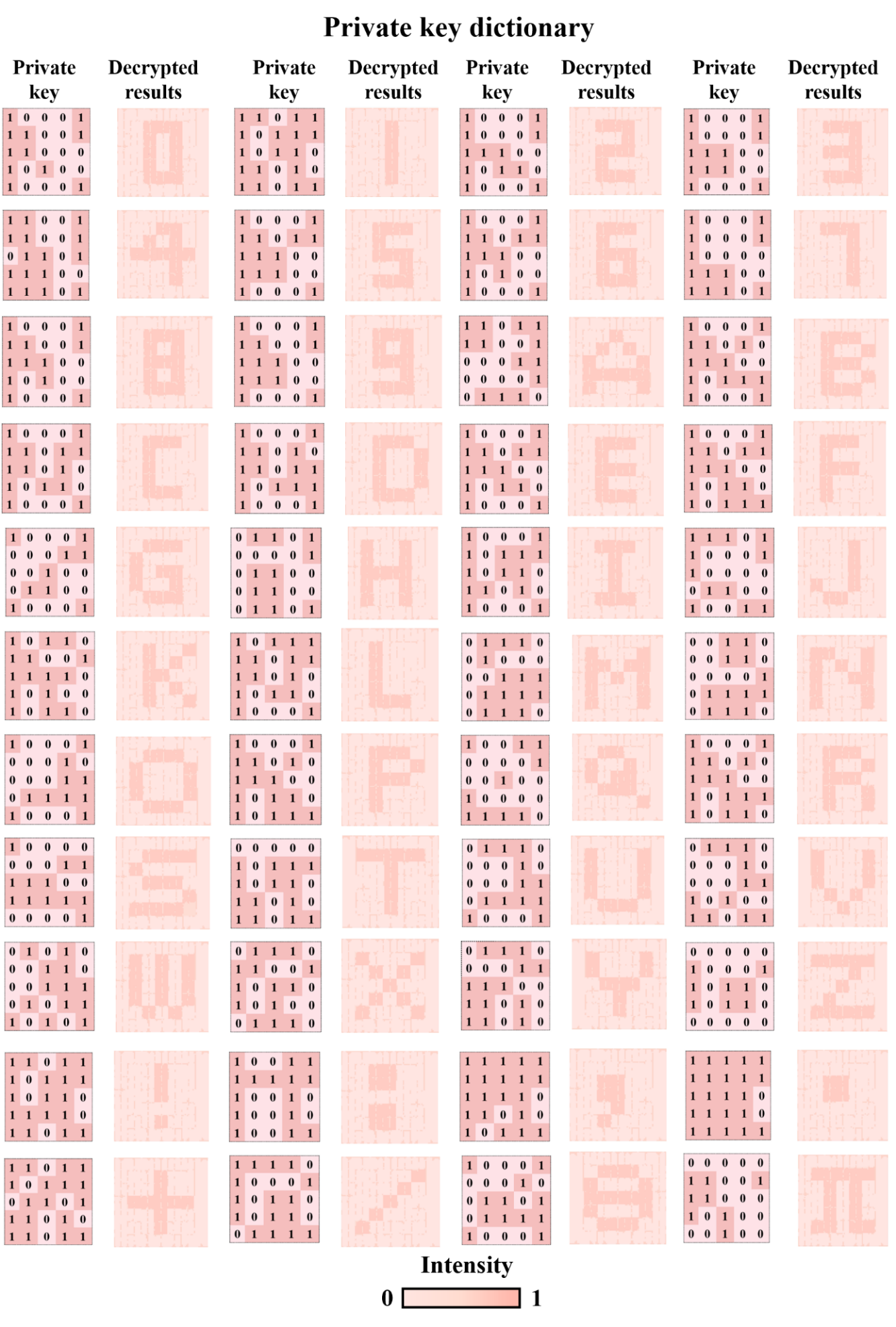


**Figure S3**. The private key dictionary and the corresponding decrypted results calculated by MATLAB.

The private key dictionary and the corresponding decrypted results calculated by MATLAB are shown in Fig. S3. We assume that Bill intends to encrypt a secret message and transmit it to Donna, and Bill needs to find the corresponding private key in private key dictionary according to the secret message.

**References**

[1] Q. Li et al., “Compact Anisotropic Metasurface for Full Range and Arbitrary Complex-Amplitude Control,” *IEEE Photonics J.*, vol. 14, no. 1, pp. 1–6, 2022.

[2] G. Ateniese, C. Blundo, A. De Santis, and D. R. Stinson, “Visual Cryptography for General Access Structures,” *Inf. Comput.*, vol. 129, no. 2, pp. 86–106, 1996.

[3] Zhi Zhou, G. R. Arce, and G. Di Crescenzo, “Halftone visual cryptography,” *IEEE Trans. Image Process*, vol. 15, no. 8, pp. 2441–2453, 2006.

[4] G. Ateniese, C. Blundo, A. D. Santis, and D. R. Stinson, “Extended capabilities for visual cryptography,” *Theor. Comput. Sci*., vol. 250, no. 1–2, pp. 143–161, 2001.

[5] R. Lukac and K. N. Plataniotis, “Digital Image Indexing Using Secret Sharing Schemes: A Unified Framework for Single-Sensor Consumer Electronics,” *IEEE Trans. on Consum.*, vol. 51, no. 3, 2005.

[6] H. Yamamoto, Y. Hayasaki, and N. Nishida, “Securing information display by use of visual cryptography,” *Opt. Lett.*, vol. 28, no. 17, pp. 1564, 2003.

[7] G. Situ and J. Zhang, “Multiple-image encryption by wavelength multiplexing,” *Opt. Lett*., vol. 30, no. 11, pp. 1306, 2005.
